# Analysis and Strategies to Improve Living Conditions of Elderly Living Alone in China: A Healthcare Context

**DOI:** 10.3390/healthcare13030219

**Published:** 2025-01-22

**Authors:** Zehao Zhang, Hongxi Di

**Affiliations:** 1 School of Public Policy and Administration, Xi’an Jiaotong University, Xi’an 710049, China; zehaoz@mail.xjtu.edu.cn; 2 College of Management, Xi’an University of Science and Technology, Xi’an 710021, China

**Keywords:** elderly living alone, elderly care services, demand, effective provision

## Abstract

**Background**: The shift toward nuclear family structures in China has resulted in a growing number of elderly individuals living alone, intensifying the imbalance between the supply and demand of elderly care services. **Objectives**: This study aims to systematically examine the care needs of elderly individuals living alone in China and propose practical strategies to enhance their quality of life. **Methods**: Using the Kano model and ERG theory, 22 care services were categorized into three types: essential (must-have), attractive, and future-focused (outlook) elements. Survey data were gathered from 230 elderly individuals living alone in Yan’an, Baoji, and Hanzhong, located in Shaanxi Province. To determine the factors influencing the intensity of demand for these services, multivariate ordinal logistic regression analysis was applied. **Results**: The findings show that demand intensity for care services is significantly shaped by factors such as gender, age, marital status, education level, income, self-rated health, loneliness, and family support. The highest demand was observed for medical and mental health services, followed by life support services. **Conclusions**: To address the gaps in elderly care services, this study suggests standardizing institutional frameworks, diversifying service options, utilizing familial support networks, and integrating intelligent technologies. These measures are especially critical for reducing service disparities in rural and less developed regions, contributing to a fairer and more effective elderly care system in China.

## 1. Introduction

With the rise in smaller family structures in China, the number of elderly individuals living alone has significantly increased, creating a growing demand for professional and socialized care services [1]. However, these elderly individuals often encounter mismatches between the supply and demand of such services, underscoring the need for collaboration among multiple stakeholders to improve service quality and accessibility. This study introduces a theoretical model and analytical framework, drawing on survey data from selected cities in Shaanxi Province, to explore the care needs of elderly individuals living alone. It also offers targeted recommendations to address these imbalances effectively.

### Theoretical Foundation and Literature Review

Drawing from Maslow’s hierarchy of needs, researcher Clayton Alderfer [2] encapsulated the characteristics and implications of the groundbreaking humanistic needs theory with the acronym ERG: E stands for existence needs, R for relatedness needs, and G for growth needs. Developing Maslow’s theory further, ERG theory proposes an intrinsic link between these different needs, suggesting that unmet higher needs can lead individuals to revert to lower-level needs.

This research used ERG theory as a framework to study the present state of elderly care demand of individuals living alone. To relate ERG theory to China’s elderly population, all elderly living alone have needs for existence, relatedness, and growth. Giranda Melani [3] studied that the group of childless elderly suffered health problems more frequently compared to others in the same age group. Due to the lack of necessary assistance for older adults living alone, they are more likely to suffer from physical and psychological difficulties, which, to some extent, makes their life satisfaction rating lower. Jinna Yu et al. [4] found that elderly people living alone tend to experience higher levels of anxiety. Their fear of loneliness, physical deterioration, depression, and isolation, as well as worries about financial hardship, may negatively affect their quality of life. Research has shown that in order to reduce anxiety in older people, the provision of comprehensive support services is essential. Such social support not only helps older people to enjoy their twilight years but also helps them to overcome psychological challenges. Haeun Park [5] and others have argued that increasing the income level of elderly people living alone and guiding them to actively participate in social interactions are essential to maintain their stable and effective quality of life. Shih Y C [6] delved into the dynamic process of elderly individuals choosing their living arrangements and indicated that living alone does not invariably result in a negative impact on their health. Shih argued that the decision to live alone arises from a multitude of factors, and reducing this choice to a mere risk factor oversimplifies the intricate processes of elderly individuals’ subjective considerations. Li Tongyao et al. [7] assessed the general health status of older adults living alone based on Chinese Longitudinal Healthy Longevity Survey (CLHLS) data, concluding that the health of this demographic is poor and requires improvement through family support and social assistance. Marit Helene Hem et al. [8] compared the mental health conditions of elderly individuals living alone and those living with others and found that the mental health of those living alone was inferior to that of the other groups, with less stable emotional states. Jenny et al. [9] showed in a European survey that people living alone need more social support and that these families must turn to social assistance to meet their social and emotional needs. Wang Bing et al. [10] conducted an analysis of the mental health status of over 1500 elderly individuals living alone in Shanghai via cluster sampling and found that individuals who were older with poor self-care abilities and lower self-rated health generally exhibited worse mental health. The researchers proposed focusing on enhancing the mental health of these individuals through cultural or sports activities, as well as social interactions. Haewon Byeon [11] notes that regular flexibility exercise for elderly people living alone is helpful in preventing depression. Numerous studies on the relationship between physical activity and depression in the community among older adults have also reported significant reductions in the incidence of depression with physical activity participation. Cheng Hongmei et al. [12] posited that social support significantly influences the life expectancy and happiness of older adults. They compared the social support received by older adults based on their living arrangements (e.g., living alone, with family, or in assisted living) and found that the level of support for those living alone was significantly lower than that for other groups.

Elderly individuals living alone will adapt to and integrate their actual circumstances and changes to their external supply to produce a graded order of needs with varying levels of intensity, which then leads to a primary, specific requirement. Donald Hedeker and his colleagues [13] conducted an empirical analysis of elderly care services in the United States, and the data indicated that as age increases and physical abilities gradually decline, the dependence of elderly individuals living alone on community-provided long-term care services rises. Zhou Rong et al. [14] found that due to economic development, financial expenditure, and other constraints, older adults living alone in second-tier cities still regard their children as the main carrier to relieve loneliness, and the intergenerational residence pattern of ‘living nearby, separated but not separated’ has an important role in alleviating the squeeze on family spiritual support and loneliness caused by residential separation. The role of intergenerational residence patterns is important in alleviating the pressure of residence separation on family spiritual support and loneliness in old age. Hana et al. [15] showed in a one-year follow-up study of elderly people living alone in Korea that social networks and support systems can help elderly people living alone to meet, integrate, and systematically obtain physical, psychological, and financial support easily and frequently using ICTs to enhance the quality of life of elderly people living alone. Mi Ran Lee [16] noted the seriousness of the issue of elderly people dying from loneliness, which constitutes a societal problem, recommending that the government should provide outdoor services and interpersonal relationship maintenance services to this group to mitigate their loneliness. Using samples of elderly individuals from communities in the Eastern, Central, and Western regions of China (according to geographic divisions), Qi Yue [17] studied the supply and demand of elderly care services for older adults living alone in each region; their findings showed that elderly individuals living alone relied more heavily on community home-based elderly care services compared to other elderly individuals and that the service needs of those living alone also differed from those of their peers. Furthermore, they found that older adults living alone had the strongest need for and highest dependence on medical and mental health care services and recommended providing targeted community home-based elderly care services based on the specific needs of older adults living alone. Yin Xingxing et al. [18] conducted OLS and Logit regression model analyses on the survey data of older adults living alone in Shanghai, Guangzhou, Chengdu, Dalian, and Hohhot and found that the supportive behaviors of older adults living alone with higher economic status were stronger, which emphasized that the current pension policy for older adults living alone should pay attention to narrowing the health gaps brought about by socio-economic status and unfolding the health-supportive policy with the family as a whole. Zhang Qi [19] used China Health and Retirement Longitudinal Study (CHARLS) data and a binary logistic model to empirically analyze the factors influencing care challenges faced by older adults living alone in China and found that familial emotional support and social support are the key factors affecting the care of elderly individuals living alone. Meanwhile, also using CLHLS data, Xiong Qian et al. [20] concluded that, unlike their peers, older adults living alone have higher demands for life support and emotional support services and suggested increasing societal attention towards older adults living alone and enhancing the recognition and fulfillment of their multi-faceted and diverse needs. Finally, Fu Haoran [21] emphasized the government’s role in purchasing elderly care services, acknowledging that while government involvement in acquiring elderly care services is both necessary and beneficial, ambiguities in the division of governmental responsibilities and the limitations of governmental capacity necessitate reliance on social entities (e.g., promoting social organizations, incorporating social funding) to effectively provide government-purchased services. Kano’s [22] Attractive Quality Theory outlines a non-linear relationship between service fulfillment and satisfaction. Applied to elderly care, it categorizes the needs of elderly individuals living alone into five types—must-be, one-dimensional, attractive, indifferent, and reverse—based on their impact on satisfaction and importance.

**Figure 1:**
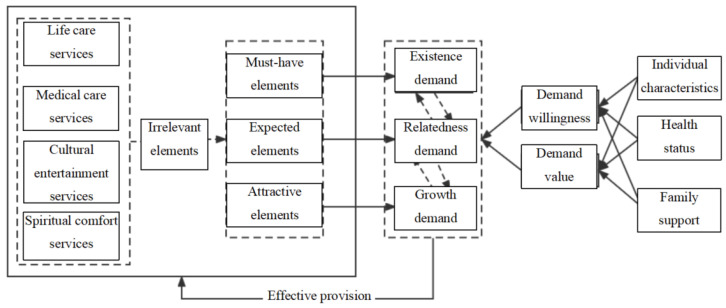
Theoretical model for elderly care service demand analysis of elderly living alone.

To summarize, this paper introduces a theoretical model and analytical framework based on ERG theory and the attractive quality theory, with a focus on elderly individuals living alone in China, as depicted in Figure 1. Existing research in this area typically begins with the concept of “demand”, categorizing elderly care services based on variations in service offerings. In contrast, the current study shifts the focus towards the actual “needs” of older adults, questioning whether the services provided truly align with their essential requirements. To achieve this, this paper applies the attractive quality theory, a well-established methodology in demand management, to classify elderly care services into three categories: must-have needs, performance needs, and attractive needs. According to the Kano model, the hierarchical structure of these services should ensure that must-have elements are met, performance elements are delivered at a high quality, and attractive elements are offered as supplementary features that may incur additional costs. In the context of ERG theory, the identified must-have services align with the “existence needs” of elderly individuals, while services that fulfill higher-level needs for relatedness and growth correspond to the “belongingness, self-esteem, and self-actualization” or “relatedness and growth” needs. It is acknowledged that elderly individuals may require services from different need levels simultaneously and that these needs may shift according to individual circumstances. Following ERG theory, after excluding irrelevant demands within current elderly care services, this study reclassifies the services to better satisfy older adults clients’ existence needs, relatedness needs, and growth needs. This paper concludes by examining the current status and emerging trends in elderly care service needs, highlighting the persistent challenges in meeting the diverse requirements of elderly individuals living alone. 

This study specifically seeks to address gaps identified in previous research by incorporating a broader geographic sample that includes central and western regions of China, as well as rural and less economically developed areas. By moving beyond the traditional focus on major coastal cities, this research provides empirical insights that offer a more comprehensive understanding of the diverse needs for elderly care services.

## 2. Materials and Methods

### 2.1. Data Source

This study centered on Shaanxi Province to examine the actual care needs of elderly individuals living alone, assess whether the current service supply meets these needs, and explore ways to ensure more effective service provision. As a region dealing with significant aging and population outflow, Shaanxi faces considerable challenges in delivering adequate care for its elderly population, especially for those living alone. As of 2021, the province’s population aged 60 and above stood at 7.6 million, making up 19.2% of the total population, which is 0.5% higher than China’s national average of 18.7%. Meanwhile, Shaanxi’s population aged 65 and above reached 5.3 million, making up 13.3% of the total, and slightly (0.2%) below the national average of 13.5%. Over 50% of the population aged 60 and above are elderly individuals living alone or in so-called “empty nests”.

In 2019, from July 21 to July 30, a survey was conducted among elderly individuals living alone in Shaanxi Province through face-to-face interviews. In Yan’an, 50 elderly participants were sampled, achieving a response rate of 85%. In Xi’an, 100 participants were sampled with a response rate of 78%, while in Baoji, 80 participants were included, with a response rate of 82%. Participants were selected using a multi-stage stratified cluster random sampling approach. First, three cities—Yan’an in the northern region, Baoji in the central region, and Hanzhong in the southern region—were selected as the first sampling layer. In the second layer, two counties or districts were chosen from each city, resulting in six counties/districts. For the third layer, two sub-district offices or townships were randomly selected from each chosen county or district, yielding twelve sub-districts/townships. Finally, in the fourth layer, two communities or institutions were randomly selected from each sub-district or township, resulting in 24 communities or institutions as the final sampling units. A comprehensive, interview-based questionnaire was then administered to each participant in their home to collect detailed data for this study. The survey collected a total of 128 interviews that took place using the “Questionnaire Concerning the High-Quality Development of Aging Services and Industry” with elderly living alone. In addition, 142 interviews took place with government and community workers, alongside the gathering of 143 supplemental data and informational resources, including policy documents, work summaries, and data charts.

### 2.2. Hierarchy of Elderly Care Service Demands of Individuals Living Alone Based on the Kano Model

#### 2.2.1. Variable Selection

Identifying the content of elderly care services is a crucial foundational step when using the Kano model to analyze the demand for such services among elderly individuals living alone. This directly influences the rationality, feasibility, practicality, and ultimate value of this research. In addition, 142 interviews were conducted with government and community workers to gather insights into the implementation of elderly care services, alongside the collection of 143 supplemental data and informational resources, including policy documents, work summaries, and data charts, which were refined to ensure they aligned with the scope of this study and provided relevant contextual support for the analysis. Elderly care service needs were categorized into four groups: life support, medical care, cultural entertainment, and spiritual comfort. The specific services included within each of these categories were as follows: meal assistance service (A1), hygiene service (A2), errand service(A3), emergency assistance (A4), security service (A5), smart elderly life service (A6), health management (A7), home diagnosis (A8), community or institutional medical service (A9), accompanying medical treatment (A10), rehabilitation nursing (A11), smart elderly medical care service (A12), elderly interest groups (A13), literary and artistic performances (A14), chess and card activities (A15), knowledge lectures (A16), smart elderly entertainment service (A17), psychological consultation and guidance (A18), accompanying chat (A19), reading out loud (A20), regular home or phone social visits (A21), and marriage agency (A22). A total of 22 service items were identified.

#### 2.2.2. Overall Analysis

A two-way question-and-answer session was conducted with each participant in their home. The results regarding older adults care service demands were then classified according to the 22 services previously identified. A total of 13 services were shown to be particularly in demand (at varying levels) by elderly individuals living alone, while the other nine original categories identified were deemed to be of interest but not of vital necessity. Of the 13 in demand services, 5 were deemed to be must-have elements, 5 were considered to be attractive elements, and 3 were categorized as outlook elements.

After categorizing all 22 services, the must-have elements included meal assistance service (A1) and emergency assistance service (A4) under life support services, health management service (A7) and community or institutional medical service (A9) under medical care services, and reading (A20) under spiritual comfort services. The attractive elements included smart elderly life service (A6), smart elderly medical care service (A12), elderly interest group (A13), literary and artistic performance (A14), and knowledge lecture (A16). The outlook elements included accompanying medical treatment (A10), psychological consultation and guidance (A18), and accompanying chat (A19). The additional but not vitally necessary services identified included hygiene service (A2), errand service (A3), security service (A5), home diagnosis (A8), rehabilitation nursing (A11), chess and card activities (A15), smart elderly entertainment service (A17), regular home or phone visits (A21), and marriage agency (A22).

### 2.3. Analysis of Factors Affecting Elderly Care Service Demands at Different Need Levels of Individuals Living Alone

#### 2.3.1. Model Setting

The explained variable in this study was the intensity of elderly care service demand, which was categorized according to a level of need and calculated within a value range from 0 to 5, with a larger number assigned to the explained variable indicating a greater intensity of demand for this type of elderly care service. Thus, the explained variable was a typical ordinal variable, and an ordinal logistic regression model was employed to analyze the key influencing factors.

The model is presented as follows:

(1) $$P=(y=m|x_{m})\frac{1}{1+\varepsilon^{\left(-\alpha +\beta x_{i}\right)}}$$

From Equation (1), an ordinal logistic regression model could be established:

(2) $$P=(y=m|x_{m})\frac{1}{1+\varepsilon^{\left(-\alpha +\beta x_{i}\right)}}$$

In Equation (2), *y* represents the likelihood of a certain intensity of elderly care service demand by elderly individuals living alone; *x* is the variable vector corresponding to the combination of characteristics that may affect demand intensity *y*; α is a constant term; *β* is the parameter vector to be estimated; and μ is the disturbance term.

1. Explained Variable

As per the previously outlined definition, demand encapsulates both the interest in and value of a particular elderly care service. Willingness regarding an elderly care service demand pertains to whether an elderly individual living alone necessitates this specific service, reflecting the presence or absence of a demand. The value of an elderly care service demand, in conjunction with the aforementioned stratification of elderly care services, refers to the number of elderly care service types this service will satisfy by elderly individuals living alone, representing the potential quantity of demand. To quantify demand intensity, any elderly care service (as defined previously) referenced by elderly individuals living alone was assigned a value of 1, while any unmentioned service was assigned a value of 0. The final sum of these values yields the index of the intensity of an elderly care service demand, with a higher total score implying a greater demand intensity for this level of elderly care service by those living alone.

2. Explanatory Variables

To better understand and reflect on the unique situations of older adults who live alone, the existing literature was reviewed extensively, and a detailed analysis was conducted of primary data from the three cities (Yan’an, Baoji, and Hanzhong) used in this study. This study places particular emphasis on the impact of family support on the demand for elderly care services among individuals living alone. To explore this, a health dimension—encompassing both physical and mental health—was introduced, considering three key perspectives: individual characteristics, health status, and family support. The individual characteristics dimension included factors such as gender, age, marital status, education level, and income. The health status dimension considered self-rated health, physical activity capacity, and feelings of loneliness. Lastly, the family support dimension encompassed aspects such as assistance with daily living, financial support, and emotional support. These dimensions provided a comprehensive framework for analyzing the factors influencing elderly care service demands.

#### 2.3.2. Research Hypotheses

1. Individual Characteristics

A review of both Chinese and international research on elderly care services highlighted that the individual characteristics of elderly individuals living alone differ significantly from those living with others. These differences lead to distinct variations in their care service needs. Key individual characteristics influencing these needs include gender, age, marital status, educational level, and income. Drawing on insights from the existing literature as well as the initial survey results and analysis, this study proposed the following research hypotheses related to individual characteristics:

**H1.**  *The individual characteristics of elderly individuals living alone play a crucial role in shaping their specific needs for elderly care services.*

**H1a.**  *Single elderly males tend to have a higher demand for elderly care services compared to single elderly females.*

Liu, Guo, and Liu [23] highlighted that gender is a key factor influencing the demand for elderly care services, with elderly males typically needing more services than their female counterparts.

**H1b.**  *Middle-aged and older individuals living alone tend to have a higher demand for elderly care services compared to their younger counterparts.*

Chen and Li [24] emphasized that age is a critical factor affecting the demand for elderly care services, with demand increasing as individuals grow older.

**H1c.**  *Widowed elderly individuals living alone exhibit a higher demand for elderly care services compared to their unmarried or married counterparts.*

Silverstein and Cong [25] observed that widowed elderly individuals receive less emotional support than their married counterparts, resulting in a higher demand for external care services.

**H1d.**  *Elderly individuals living alone with a higher level of education tend to have a greater demand for elderly care services compared to those with a lower level of education.*

Chen and Liu [26] demonstrated that educational level significantly impacts elderly individuals’ health awareness and demand for care services, with those possessing higher levels of education being more inclined to seek professional care services.

**H1e.**  *Elderly individuals living alone with higher incomes tend to have a greater demand for elderly care services compared to those with lower incomes.*

Feng and Glinskaya [27] demonstrated that income level plays a significant role in influencing the demand for elderly care services, as higher-income individuals have both the financial means and a greater interest in accessing higher-quality care services.

2. Health Status

Based on the preliminary survey results and analysis, the following research hypotheses regarding health status were proposed:

**H2.**  *The health status of elderly individuals living alone plays a significant role in shaping their demand for elderly care services.*

**H2a.**  *Elderly individuals living alone who rate their health as poor exhibit a higher demand for elderly care services.*

Zeng and Chen [28] found that elderly individuals with poorer self-rated health are more likely to seek and utilize care services.

**H2b.**  *Among elderly individuals living alone, the number of chronic diseases significantly influences their demand for elderly care services, with those managing more chronic conditions exhibiting a higher demand for such services.*

Zhang and Li [29] demonstrated that the presence of chronic diseases substantially increases the demand for medical care services among elderly individuals.

**H2c.**  *Loneliness significantly impacts the demand for elderly care services among individuals living alone, with those experiencing higher levels of loneliness exhibiting a greater need for such services.*

Hawkley and Cacioppo [30] highlighted that loneliness is a significant predictor of elderly individuals’ demand for care services, with a greater sense of loneliness correlating with a higher demand for such services.

3. Family Support

Family support refers to the assistance provided to elderly individuals living alone by their family members, particularly their children. This support can be categorized into three main types: financial support, life support, and emotional support. Based on the survey results and analysis, the following research hypotheses were proposed regarding family support:

**H3.**  *The level of family support significantly influences the demand for elderly care services among elderly individuals living alone.*

**H3a.**  *Elderly individuals living alone who receive greater financial support from their families tend to exhibit a higher demand for elderly care services compared to those receiving less financial support.*

**H3b.**  *Elderly individuals living alone who receive substantial life support from their families tend to have a lower demand for elderly care services compared to those who receive less family life support.*

**H3c.**  *Elderly individuals living alone who receive greater emotional support from their families tend to have a lower demand for elderly care services compared to those who receive less emotional support.*

## 3. Results

### 3.1. The Hierarchy of Elderly Care Service Needs of Individuals Living Alone Based on the Kano Model

#### 3.1.1. Overall Analysis

The Kano model was applied to evaluate the demand for elderly care services among individuals living alone, providing not only a classification of services but also a deeper analysis of their specific characteristics. Demand levels vary depending on how critical each service is perceived. Must-have elements (M) are essential services that directly impact quality of life and are non-negotiable. Attractive elements (A) enhance satisfaction but are not strictly necessary, offering additional benefits. Outlook elements (O) focus on future development or potential needs, adding value without being immediate necessities. This approach helps prioritize services based on their relevance and importance to older adults living alone.

This analysis delves deeper into the implications of these service classifications. For instance, **meal assistance (A1)** and **emergency assistance (A4)** are categorized as must-have elements because they meet essential survival needs. In contrast, services like **literary and artistic performances (A14)** are classified as attractive elements, as they enhance life satisfaction without directly affecting daily living. By distinguishing these categories, policymakers and service providers can allocate resources more effectively, focusing on services that have the greatest impact on the well-being of elderly individuals living alone.

#### 3.1.2. Better–Worse Satisfaction Coefficient Analysis

**Figure 2:**
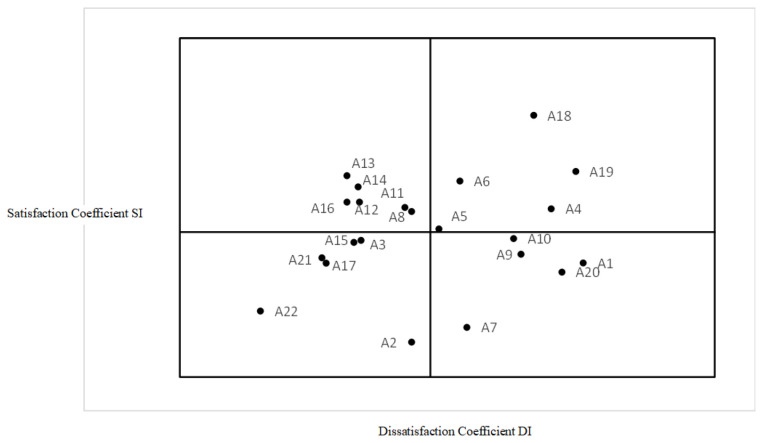
Four-quadrant diagram presenting elderly care service better–worse coefficient values.

The **best-worst ratio analysis** was employed to evaluate the relative importance of various factors by comparing the highest (best) and lowest (worst) scores assigned by participants. This method provides a clear ranking based on participants’ preferences. The better–worse coefficients for each service were calculated from survey responses by elderly individuals living alone, specifically analyzing their satisfaction when a service was provided (“better”) versus when it was not provided (“worse”). The average absolute values of the “better” and “worse” coefficients across all surveyed services were 0.428 and 0.468, respectively. These values were used as the horizontal and vertical origin coordinates, forming the basis for a four-quadrant diagram of the 22 elderly care services (see Figure 2). This visualization aids in identifying and prioritizing services based on their perceived significance and impact. The first quadrant contains outlook elements, the second quadrant attractive elements, the third quadrant non-essential but beneficial elements, and the fourth quadrant essential elements. Analyzing the magnitude of these values can help in the prioritization of service demands, as a higher value suggests that this service is of greater necessity to elderly individuals living alone, indicating that the service should be prioritized to fulfill their needs.

#### 3.1.3. The Hierarchy Model of Elderly Care Service Needs of Individuals Living Alone

The Kano model delineates the multi-tiered and stratified characteristics of service requisites among the geriatric population, facilitating the stratification of their demands into fundamental, performance-oriented, and aspirational categories. Concurrently, the ERG theory augments this framework by elucidating the diverse needs of older adults through its emphasis on the dimensions of existence, relatedness, and growth, which are similar to the complex and changing nature of their care needs. The synthesis of these two theoretical constructs provides a robust analytical foundation for dissecting the multi-dimensional needs of solitary elderly individuals, thereby ensuring a comprehensive apprehension of their caregiving demands.

**Figure 3:**
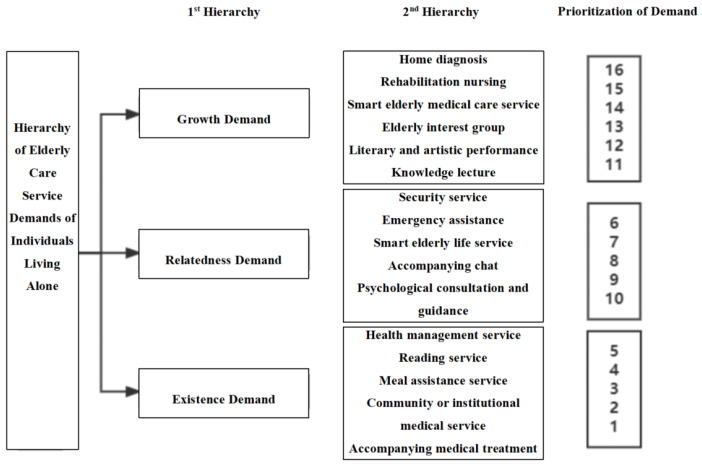
Hierarchy model of elderly care service demands for individuals living alone based on ERG theory.

In accordance with this analytical framework and the precepts of the ERG theory, the foundational elements of geriatric care services were categorized under the rubric of existence needs, the socio-relational components under the purview of relatedness needs, and the aspirational facets under the category of growth needs. After excluding the non-essential elements (i.e., services of little interest or with low uptake by individuals living alone), the order of prioritization of the remaining elderly care services was “must-have elements > outlook elements > attractive elements”. Within each need category, the larger the coefficient value of the service, the higher its priority. The hierarchy model for elderly care service demands of individuals living alone is thus depicted in Figure 3.

### 3.2. Analysis of the Factors Affecting Elderly Care Service Demands Within the Different Hierarchy Levels

#### 3.2.1. Model Fit Test

The application of a multivariate ordinal logistic regression model in this study was based on two hypothesis conditions: the parallel line tests and the overall model’s validity. SPSS 22 statistical software was used to perform these tests, including model fit assessments for the three-level model of elderly care service demands, using data from 128 interviews. The results showed that all three models—existence-oriented (Model 1), relatedness-oriented (Model 2), and growth-oriented (Model 3)—exhibited a good fit, with all fitting indicators meeting the hypothesis test requirements. In the parallel line test, the *p*-values for Model 1, Model 2, and Model 3 were 0.834, 1.000, and 1.000, respectively, all exceeding the 0.05 threshold. These results confirm that the use of ordinal logistic regression in this analysis was valid. Furthermore, the findings were statistically significant, and the model demonstrated strong explanatory power for understanding elderly care service demands.

#### 3.2.2. Regression Results

The multivariate ordinal logistic regression analysis identified key factors influencing the intensity of elderly care service demands among individuals living alone, with the results presented in **Table 1**. All independent variables included in the model were found to have a significant impact on the dependent variable. The most significant positive factors influencing demand intensity were, in descending order, the number of chronic diseases, loneliness, family financial support, and family life support. In contrast, the factors with the most significant negative impact on existence-oriented elderly care service demands were gender (with females as the reference group) and self-rated health status. These findings highlight the critical role of health conditions and family support in shaping elderly care service needs for individuals living alone.

1. Impact of Individual Characteristics on Elderly Care Service Demands of Individuals Living Alone

For individual characteristics, gender (with females as the reference) significantly and negatively impacted all three levels of elderly care service demands of individuals living alone. This indicated that elderly males living alone exhibit higher demands for elderly care services and are more strongly dependent than their female counterparts. Meanwhile, age was inversely correlated with elderly care service demands, suggesting that the older the individual, the lower their dependence on elderly care services. Contrary to younger elderly individuals, who are more independent and have greater mobility, middle-aged and older elderly individuals tend to have less demand for services. Interestingly, this result diverges from those of previous studies focused on older adults living alone. Prior research has generally concluded that, as age increases and physical function declines, the dependence of elderly individuals living alone on various types of elderly care services tends to grow. This discrepancy may be the result of the sample data used in the current study, as the participants interviewed in this study were primarily self-sufficient or semi-dependent, with only a limited number of disabled elderly included in the sample. Among elderly individuals with a higher level of self-care ability, those with a higher level of energy reported being more active and more inclined to utilize social elderly care services.

2. Impact of Health Status on Elderly Care Service Demands of Individuals Living Alone

The analysis results regarding health status indicated that self-rated health positively impacted the level of service demand, with chronic diseases significantly and negatively affecting elderly care service demands, while loneliness also exerted a significantly positive effect on existence-oriented elderly care service demands. Altogether, it can be inferred that elderly individuals living alone with a lower level of health (i.e., those reporting poor self-rated health and more chronic diseases) demonstrated higher demands for elderly care services. Elderly individuals living alone who frequently experienced loneliness also exhibited a higher demand for services. The regression results affirm that elderly individuals living alone with good health are more inclined to be self-reliant. When they are capable of caring for themselves, some may prefer to stay at home and care for themselves, demonstrating a lower reliance on various types of elderly care services but having higher expectations of the services they employ. Conversely, elderly individuals living alone with poor health, burdened by subpar physical and/or psychological conditions and finding themselves in a solitary living situation, require more social care support, relying heavily on elderly care services while having lower expectations of the services received.

3. The Impact of Family Support on older adults’ Care Service Demands of Individuals Living Alone

While all three types of family support (i.e., financial support, daily living assistance, and emotional support) passed the significance test, it is noteworthy that, in older adults care service model, family financial support and family daily living assistance exerted a significant positive influence on service demand, while family emotional support had a significant negative impact. Using the existence-oriented elderly care service model (Model 1) as an example, the intensity of existence-oriented elderly care services for individuals living alone who are receiving financial and life support from their family escalated by 14.222 times and 4.455 times, respectively. The regression coefficient value for emotional support was −2.387, indicating significance at the 0.01 level (z = −4.913, *p* = 0.000 < 0.01), implying that emotional support has a significantly negative impact on the demand for existence-oriented elderly care services. In other words, the less emotional support one receives from family when living alone, the higher their dependence on existence-oriented elderly care services. This suggests the need to segment family-provided support, as the impact of various types of family support on the demand for social elderly care services for individuals living alone will vary.

Table 1 presents the regression coefficients (z-scores) and *p*-values, with larger z-scores indicating stronger effects and *p*-values below 0.05 signifying statistical significance. The analysis, as detailed in **Table 1**, underscores the validity of the model and the impact of various variables on the demand for elderly care services. Among individual characteristics and health factors, gender was not found to have a significant impact on any care service category. However, being widowed substantially increased the demand for both existence-oriented and growth-oriented services. Higher education levels were associated with a significant demand for existence-oriented and relatedness-oriented care, while income notably influenced the demand for growth-oriented care services. In terms of health, self-rated health showed an inverse relationship with service demand, whereas the number of chronic conditions and feelings of loneliness positively correlated with the demand for all service types. Additionally, financial and life support from family members significantly boosted the demand for all elderly care services, with a pronounced effect on existence-oriented and relatedness-oriented services. Overall, family support, health status, and social support emerged as pivotal factors shaping the demand for elderly care services, with their influence varying across different service categories.

## 4. Discussion

### 4.1. The Hierarchy of Elderly Care Service Demands of Individuals Living Alone Based on the Kano Model

The current study’s findings on the hierarchy of elderly care services align with those of the existing literature on the evolving needs of older adults population. As a demographic age, it will exhibit increasingly diverse patterns in living arrangements. For instance, the higher demand for medical services found in this study, particularly in terms of accompaniment for medical treatment, mirrors findings from similar studies conducted in other aging societies, such as Japan and the Republic of Korea, where the necessity for health care support is increasingly recognized as being fundamental for older adults living alone [31,32]. The variation observed in the current findings in the demand for services such as meal assistance and health management is also in line with the findings of studies emphasizing the critical nature of such services in improving and maintaining the daily life experiences of elderly individuals living alone [33].

### 4.2. Analysis of the Factors Affecting Elderly Care Service Demand in the Different Hierarchies of Needs of Individuals Living Alone

The need to regulate the service system for elderly care, particularly with individuals living alone in mind, has been supported by research internationally, highlighting the importance of comprehensive legal frameworks and long-term planning in addressing the needs of aging populations [34]. The structural challenges observed in the provision of elderly care services in China are not unique; similar issues have been documented in studies from Europe and North America. These regions are increasingly exploring mixed models that combine government and market-based solutions to optimize the delivery of elderly care services [35,36].

### 4.3. Encouraging the Participation of Social Forces in Diversified Supply

The integration of government and market mechanisms to enhance elderly care service provision aligns with findings from the literature on public–private partnerships in elderly care. Studies from the United States and Germany have shown that such collaborations can lead to more effective and responsive care services, particularly benefiting elderly individuals living alone [37,38]. Similarly, the study in Shaanxi, China, revealed that providing adequate social support to older adults promotes improvements in their mental health and significantly enhances their sense of self-worth and value [39]. These findings highlight the importance of policy innovation and the inclusion of social forces to diversify elderly care service providers and address the evolving needs of this population.

### 4.4. Accurately Identifying Effective Elderly Care Service Demand

Our findings underscore the importance of a unified and standardized demand evaluation system for accurately assessing and addressing the needs of elderly individuals living alone. This aligns with international studies emphasizing the necessity of personalized care approaches to effectively meet the specific needs of this demographic [40]. Additionally, the implementation of dynamic family support evaluation mechanisms is supported by the existing literature, which highlights their critical role in tailoring social and family-based services to the actual needs of elderly individuals [41]. In addition, a Korean study showed that there are gender differences in physical health, mental health, and cognitive functioning of elderly people living alone in terms of identifying the need for elderly care services, which need to be personalized based on gender [42].

### 4.5. Promoting the Culture of Family Elderly Care

The promotion of family-based elderly care as a strategy to enhance support aligns with findings from cultures that traditionally emphasize filial piety, such as the Republic of Korea and Taiwan. Studies in these regions have demonstrated that robust family support systems can significantly reduce the reliance of elderly individuals on public care services while enhancing the quality of life for those living alone [43,44]. Policy initiatives supporting family-based care, including care allowances and leave policies, have already been successfully implemented in several countries and could serve as valuable models for similar policies in China.

### 4.6. Strengthening Older Adults’ Care Service Workforce

The need to strengthen the workforce in elderly care services is a consistent theme in global studies on aging populations. Research from Canada and the United Kingdom has shown that improving the social status and professional development of elderly care workers is essential for enhancing service quality [45,46]. The findings of this study reinforce these conclusions, highlighting the critical role of a well-trained and fairly compensated workforce in delivering high-quality care to elderly individuals living alone.

### 4.7. Innovating Technological Applications

The role of technology in elderly care has been extensively discussed in the literature, with studies from Nordic countries demonstrating the potential of smart technologies to enhance care provision [47,48]. A comparative study in South Korea highlighted the effectiveness of social robot interventions in improving cognitive function and reducing depression and loneliness among older individuals living alone [49], underscoring the transformative impact of innovative technologies in elderly care. The findings of this study point to similar opportunities in the Chinese context, suggesting that technological innovations could significantly bridge service gaps, particularly for elderly individuals living alone. For instance, telemedicine services can provide remote health consultations and monitoring, enabling elderly individuals to access medical advice without leaving their homes. Furthermore, smart home technologies equipped with emergency response systems and daily activity monitoring can enhance safety and deliver timely assistance during accidents or health issues. However, as noted in other studies, it is essential to ensure that these technologies are accessible and user-friendly, especially for elderly populations [50]. Accessibility and ease of use will be critical in maximizing the potential benefits of these technological solutions.

The contradictory findings regarding age and the demand for elderly care services warrant further investigation. While prior research generally associates aging with increased care needs, our study identifies a negative correlation between age and service demand. This discrepancy may stem from the composition of our sample, which predominantly includes elderly individuals with strong self-care abilities who report lower service needs compared to more vulnerable groups. Additionally, confounding factors such as health status, social engagement, and income may interact with age, playing a significant role in shaping service demand. Furthermore, cultural and societal perceptions of aging and elder care could influence patterns of service utilization, leading to variations across different contexts. These complexities highlight the need for more nuanced research to better understand the relationship between age and care service demand.

These findings underscore the limitations of this study, as it was conducted in specific regions of China, which may restrict the generalizability of the results. Future research should address these limitations by expanding the sample to include elderly populations from a broader range of geographical and socio-economic backgrounds and by conducting cross-regional comparative studies. Furthermore, adopting longitudinal research designs would enable the tracking of how care needs evolve over time with changes in age and other factors, offering deeper insights into the dynamic nature of care requirements. Stratified sampling based on functional ability and analyzing interactions between socio-demographic factors could further enhance the understanding of this complex relationship.

## 5. Conclusions

This study investigated older adults’ care service demands of individuals living alone, drawing on field research conducted in Yan’an, Baoji, and Hanzhong, Shaanxi Province, China. Utilizing the Kano model, this research identified 16 essential services, categorized as must-have, attractive, or outlook elements, and developed a hierarchy of demand priorities. The findings highlight that elderly individuals living alone have a significant reliance on medical and mental health services, with life support services being the next most important category.

The analysis revealed that the demand for elderly care services is shaped by individual characteristics (e.g., gender, age, marital status, education, and income), health status (e.g., self-rated health and mental health), and family support (e.g., financial and life support). These findings highlight the importance of implementing targeted strategies, such as conducting health status assessments, supplementing family support, and enhancing social care services, particularly for widowed elderly individuals who lack sufficient emotional support.

Despite notable progress, elderly care services in China continue to face significant challenges, including structural imbalances in supply, limited service capacity, and accessibility gaps, particularly in rural areas. To address these issues, coordinated efforts are essential to standardize legal frameworks, promote a diversified supply of services, strengthen workforce capacity, and integrate smart technologies. These strategies aim to create a more responsive and sustainable elderly care system, ultimately enhancing the quality of life for elderly individuals living alone.

## Figures and Tables

**Table 1:** Regression results of elderly care service demands of elderly individuals living alone.

Dimensions	Independent Variables	Existence-Oriented Elderly Care Services (Model 1)	Relatedness-Oriented Elderly Care Services (Model 2)	Growth-Oriented Elderly Care Services (Model 3)
Individual Characteristics	Gender	−2.327 ** (0.098)	−2.274 ** (0.103)	−1.444 * (0.236)
	Aged 70–79	−2.268 ** (0.103)	−1.189 * (0.304)	−0.811 (0.444)
	Aged 80 and above	−2.921 ** (0.054)	−1.634 ** (0.195)	−1.147 * (0.318)
	Widowed	3.352 ** (0.035)	2.738 ** (0.065)	4.436 ** (0.012)
	Junior High School	−2.045 ** (0.129)	−1.301 * (0.272)	−2.228 ** (0.108)
	High School and above	2.027 ** (7.595)	1.746 ** (5.734)	1.069 (2.912)
	Income	0.398 * (1.489)	0.293 * (1.341)	0.778 ** (2.178)
Health Status	Self-Rated Health	−0.577 * (1.781)	−0.501 * (1.651)	−0.646 * (1.908)
	Number of Chronic Diseases	0.917 ** (0.400)	0.631 * (0.532)	0.640 * (0.528)
	Loneliness	0.214 ** (1.239)	0.138 ** (1.147)	0.263 ** (1.301)
Family Support	Financial Support	2.655 ** (14.222)	2.073 ** (7.952)	1.045 * (2.845)
	Life Support	1.494 ** (4.455)	1.370 ** (3.936)	1.111 * (3.037)

Note: * *p* < 0.05, ** *p* < 0.01; OR values are shown in parentheses.

## Data Availability

Data are contained within this article.
